# Renin-Angiotensin System Blockade and Mortality in Patients With Hypertension and COVID-19 Infection

**DOI:** 10.1177/1074248420947628

**Published:** 2020-08-04

**Authors:** Husam M. Salah, Guisseppe Calcaterra, Jawahar L. Mehta

**Affiliations:** 1 Department of Medicine, University of Arkansas for Medical Sciences, Little Rock, AR, USA; 2 Post Graduate Medical School, University of Palermo, Palermo, Italy

**Keywords:** COVID-19, renin-angiotensin-aldosterone system, hypertension

## Abstract

To determine the effect renin-angiotensin system blockers on the outcome in patients with hypertension and concurrent COVID-19 infection, we searched PubMed, the Cochrane Library, and Google Scholar for relevant articles. Twelve studies with a total of 16,101 patients met the inclusion criteria. The mortality rate among the users of angiotensin converting enzyme inhibitors or angiotensin receptor blockers was 12.15% and in non-users it was 14.56% (risk ratio 0.70, 95% CI [0.53-0.91], P < 0.007). There was no difference in the risk of death between the use of angiotensin converting enzyme inhibitors and angiotensin receptor blockers (risk ratio 1.09, 95% CI [0.90 -1.32]). We conclude that the use of angiotensin converting enzyme inhibitors and angiotensin receptor blockers improves mortality in patients with hypertension and concurrent COVID-19 infection, without a significant difference between ACEIs and ARBs in this population.

## Introduction

Coronavirus disease 2019 (COVID-19), also known as severe acute respiratory syndrome coronavirus-2 (SARS-CoV-2), is an ongoing pandemic and a major healthcare concern. SARS-CoV-2 requires angiotensin-converting enzyme ^2^ (ACE2) protein to enter ACE2-expressing cells.^1^ ACE-2 is a component of renin-angiotensin system (RAS) activation which plays an important role in hypertension. This association between ACE2 and SARS-CoV-2 stimulated interest in examining the relationship between RAS inhibitors and COVID-19 infection.

It has been proposed that upregulation of ACE,^2^ as observed in hypertension, may be the basis of higher COVID-19-related mortality in hypertensive subjects. This postulate also prompted studies on the effect of the ACE inhibitors (ACEIs) and angiotensin receptor blockers (ARBs) on outcome in patients with COVID-19 infection. Studies have reported conflicting results regarding the effect of ACEIs and ARBs on the overall outcome in patients with COVID-19 infection. A pooled analysis of 6 studies showed no statistically significant association between ACEI/ARB use in patients with COVID-19 and mortality.^2^ However, this study did not specifically examine this association in patients with hypertension and concurrent COVID-19 infection. Feng et al^3^ suggested that the use of ACEIs/ARBs in patients with hypertension and concurrent COVID-19 infection can reduce mortality. On the other hand, Khera et al^4^ showed no significant difference in mortality between patients with hypertension and COVID-19 receiving ACEIs/ARBs and those who were receiving other anti-hypertensive agents. Small meta-analyses showed lower risk of mortality in patients with concurrent COVID-19 and hypertension who were on ACEI/ARB compared to those who were not.^5-7^

In this study, we aimed to investigate the effect of RAS inhibition in patients with hypertension and concurrent COVID-19 infection. Further, we studied the relative effects of ACEIs and ARBs in patients with hypertension and concurrent COVID-19 infection.

## Methods

PubMed, the Cochrane Library, and Google Scholar were searched to collect results of studies on the outcomes of users and non-users of ACEIs and ARBs in hypertensive patients with concurrent COVID-19 infection between the 1st of January of 2020 and the 16th of June of 2020. There was no language restriction placed in the literature search. The following terms were used in search: “COVID-19” and “angiotensin-converting enzyme inhibitors,” “COVID-19” and “ACE inhibitors,” “COVID-19” and “angiotensin II receptor blockers,” “COVID-19” and “ARB,” and “COVID-19” and “ARBs.” A Google search was also performed. The inclusion criteria were as follows: (1) Study population included patients with COVID-19 infection with concurrent hypertension; (2) Only Cohort studies, case-control studies, and case series studies were included; (3) Mortality rate was reported or could be calculated using the provided data. All studies that had patients with COVID-19 infection without detailed information on hypertension and specific use of RAS inhibitors were excluded

There were 2 groups of patients in our study; the first group included patients with confirmed hypertension and concurrent COVID-19 infection who were taking ACEIs or ARBs, and the second group included patients with hypertension and confirmed COVID-19 infection who were not taking ACEIs or ARBs. The analysis was performed using the Review Manager 5.4 software. The primary planned outcome was the risk ratio between the 2 groups. We used a random effect model to analyze the pooled data. The risk ratio between the 2 groups was reported with 95% confidence interval (95% CI). The Chi-squared statistic, its degrees of freedom (df), and the I^2^ index were used as measures of heterogeneity. Funnel plots were included to assess for potential publications bias. We then performed another meta-analysis to compare mortality between patients with hypertension and concurrent COVID-19 infection who were receiving ACEIs and those with hypertension and concurrent COVID-19 infection who were receiving ARBs.

## Results

**Figure 1.**
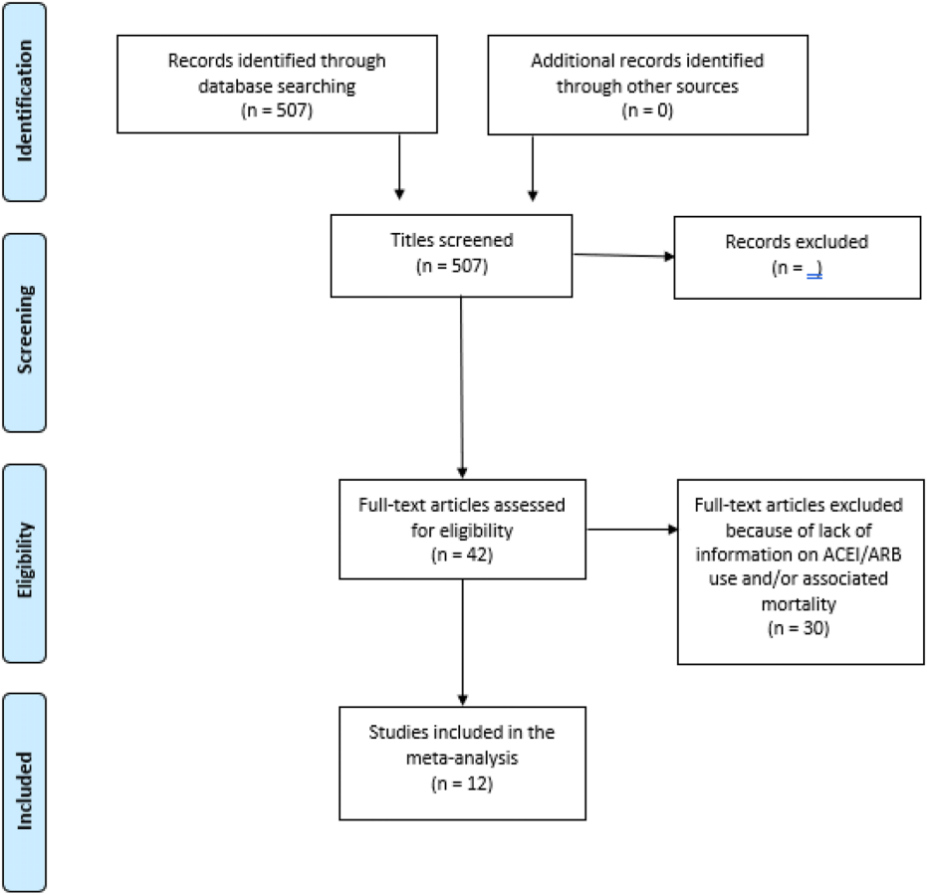
Flow PRISMA diagram for the study inclusion and exclusion.

**Figure 2.**
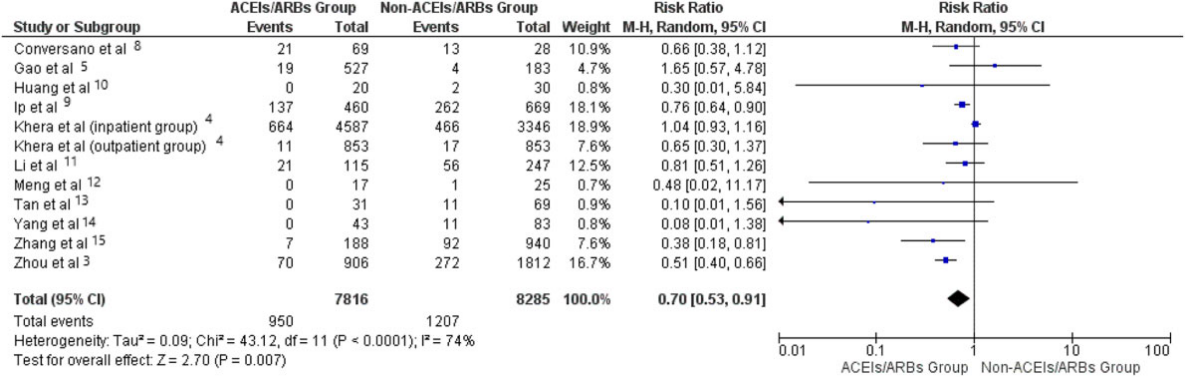
Forest plot comparing mortality between patients with hypertension and concurrent COVID-19 infection who were receiving ACEIs/ARBs and those who were not.

**Figure 3.**
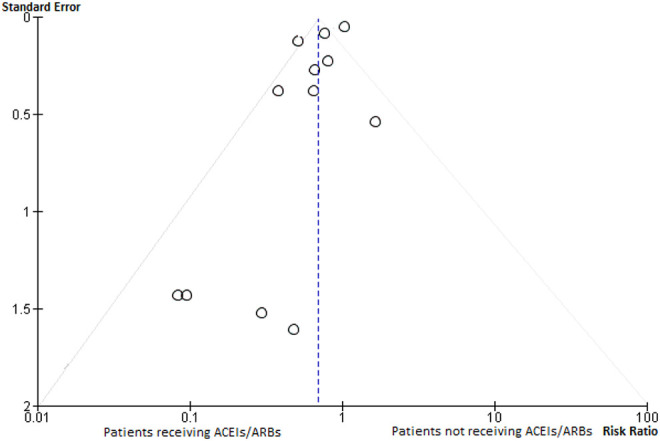
Funnel plot assessing publication bias for studies comparing mortality between patients with hypertension and concurrent COVID-19 infection who were receiving ACEIs/ARBs and those who were not.

A total of 507 studies were initially identified. Only, 12 studies^3-5,8-15^ met the strict inclusion criteria with a total of 16,101 patients with hypertension and concurrent COVID-19 infection (Figure 1). Of those, 7816 patients were taking ACEIs or ARBs. Although there was some variability in the effect of RAS inhibitors on mortality, the pooled data showed that mortality rate among the ACEIs/ARBs users was 12.15%, whereas mortality rate among the non-users was 14.56% (risk ratio—0.70, 95% CI 0.53-0.91], P < 0.007). I^2^ index was 76% (Figures 2 and 3). Sensitivity analysis showed consistent results (Table 1).

**Table 1.** Sensitivity Analysis of the Studies Comparing Mortality Between Patients With Hypertension and Concurrent COVID-19 Infection Who Were Receiving ACEIs/ARBs and Those Who Were Not.

	RR	95% CI
Removal of the 3 studies with the biggest weight	0.64	[0.45,0.92]
Removal of the 3 studies with the lowest weight	0.73	[0.56,0.94]

**Figure 4.**
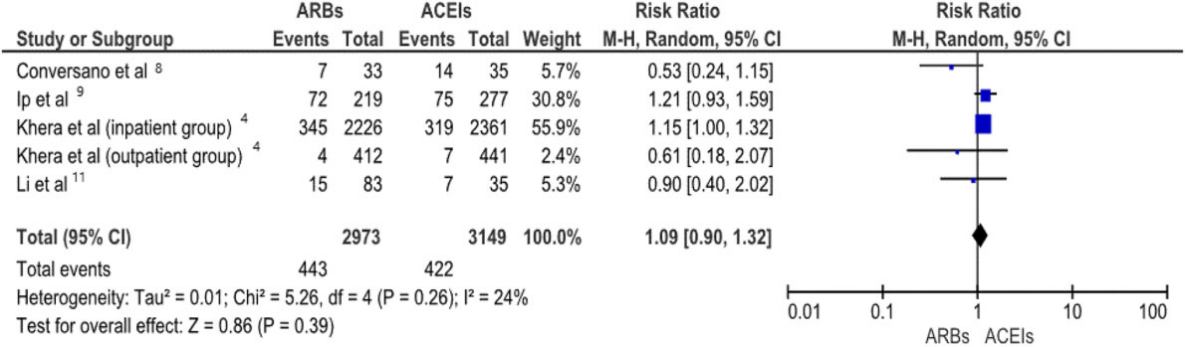
Forest plot comparing mortality rates between patients with hypertension and concurrent COVID-19 infection who were receiving ACEIs or ARBs in a random effect model.

**Figure 5.**
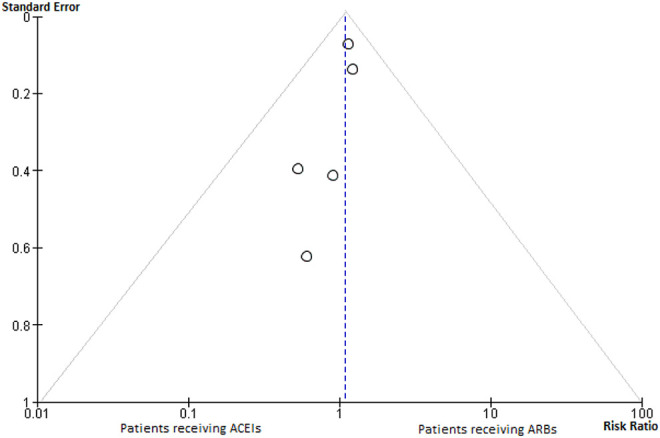
Funnel plot assessing publication bias for studies comparing mortality between patients with hypertension and concurrent COVID-19 infection who were receiving ACEIs or ARBs.

For comparison of mortality rates between ACEIs and ARBs users, only 5 of the initial 11 studies qualified with a total of 6122 patients. The other studies did not have details on the mortality rates in ACEIs in comparison to ARBs. The analysis showed no difference between the use of ACEIs and ARBs on the risk of death (risk ratio 1.09, 95% CI [0.90 -1.32]). I^2^ index was 24% (Figures 4 and 5). Sensitivity analysis showed consistent results (Table 2).

**Table 2.** Sensitivity Analysis of the Studies Comparing Mortality Between Patients With Hypertension and Concurrent COVID-19 Infection Who Were Receiving ACEIs Versus ARBs.

	RR	95% CI
Removal of the 2 studies with the biggest weight	0.67	[0.40,1.12]
Removal of the 2 studies with the lowest weight	1.10	[0.87,1.39]

## Discussion

Our analysis provides definitive evidence that inhibition of RAS provides survival benefits in patients with hypertension and concurrent COVID-19 infection. Our analysis also shows no statistically significant difference in mortality rates between users of ACEIs and users of ARBs.

The pathophysiological mechanism underlying the beneficial effect of ACEIs and ARBs is not understood. ACE2 activation has vasodilator properties, and as such a loss of ACE2 expression and function could lead to the development of hypertension.^16^ Although not conclusively shown, it is possible that RAS inhibition enhances tissue ACE2 expression and function, and ACE2 upregulation by ACEIs/ARBs might explain the survival benefit associated with their use is patients with hypertension and concurrent COVID-19 infection.^16^ Sama et al^17^ recently showed a reduction in plasma ACE2 levels in a large number of heart failure patients receiving ACEIs/ARBs. However, relationship between plasma ACE2 levels and tissue ACE expression has not been studied in COVID-19 patients, especially those with hypertension. Until we know about the relative expression of ACE2 and its role in patients with hypertension and COVID-19 infection, the modulation of ACE2 by ACEIs/ARBs remains a matter of conjecture.

## Conclusions

Our analysis shows that the use ACEIs and ARBs improves mortality in patients with hypertension with concurrent COVID-19 infection. There is no difference in mortality between ACEIs and ARBs in this population.
